# Kinome-wide CRISPR-Cas9 screens revealed *EXOSC10* as a positive regulator of TGF-β signaling

**DOI:** 10.1016/j.bbrep.2024.101864

**Published:** 2024-11-12

**Authors:** Dingding Wang, Xinhao Zhang, Jianxun Guo, Weijia Liu, Yanchi Zhou, Renxian Wang

**Affiliations:** a JST Sarcopenia Research Centre, National Center for Orthopaedics, Beijing Research Institute of Traumatology and Orthopaedics, Beijing Jishuitan Hospital, Capital Medical University, Beijing, 100035, China; b Department of Biochemistry and Molecular Biology, Institute of Basic Medical Sciences Chinese Academy of Medical Sciences, School of Basic Medicine Peking Union Medical College, Beijing, 100005, China; c Center for Bioinformatics, Institute of Basic Medical Sciences, Chinese Academy of Medical Sciences, School of Basic Medicine, Peking Union Medical College, Beijing, 100005, China; d Department of Hepatobiliary Surgery, National Cancer Center/National Clinical Research Center for Cancer/Cancer Hospital, Chinese Academy of Medical Sciences and Peking Union Medical College, Beijing, 100021, China

**Keywords:** TGF-β signaling, *EXOSC10*, Kinome-wide CRISPR screen

## Abstract

The TGF-β signaling pathway is closely associated with human health and disease, and the systematic identification of factors involved in the TGF-β signaling pathway significantly contributes to the understanding and treatment of various diseases. Through kinome-wide CRISPR screen, we identified 13 candidate regulatory targets. Notably, the well-known hallmark genes *TGFBR1* and *TGFBR2* emerged as the top two candidate targets. *OXSR1* and *EXOSC10* were ranked third and fourth as positive candidate targets, respectively, with *EXOSC10* being a novel discovery. Importantly, our findings revealed the down-regulation of *OXSR1* and *EXOSC10* using CRISPR knockout and RNAi technology effectively suppressed the TGF-β signaling pathway in HeLa and HaCaT cells, providing new insights of TGF-β signaling.

## Highlights

• Kinome-wide CRISPR knockout screen systematically identified genes involved in the TGF-β signaling pathway.
• A total of 13 candidate targets were identified, with hallmark genes *TGFBR1* and *TGFBR2* ranking as the top two.
• Down-regulation of *OXSR1* and *EXOSC10* effectively suppressed the TGF-β signaling pathway.

## 1 Introduction

Transforming growth factor-β (TGF-β) signaling plays a crucial role in cell proliferation, apoptosis, migration, differentiation, immune regulation, and extracellular matrix remodeling across various cell types [1,2]. Dysregulated TGF-β signaling transductions are often closely associated with the development of cancer, cardiovascular diseases, tissue fibrosis, and skeletal disorders [[2], [3], [4], [5]]. A comprehensive understanding of the TGF-β signaling pathway can help unravel the mechanisms underlying the development and treatment of diseases such as cancer.

Being the primary method for high-throughput functional genomics screen, the CRISPR-Cas9 system plays a crucial role in identifying mechanism and targets of various diseases owing to its simplicity, speed, high efficiency, and accuracy in achieving gene editing [[6], [7], [8]]. Protein kinases act as pivotal regulatory factors in modulating the TGF-β signaling pathway. For example, the TGF-β ligand binds to the serine-threonine kinase TβRII, which subsequently recruits TβRI, another serine-threonine kinase, to forms a ligand-receptor complex that phosphorylates downstream SMAD signaling proteins [9,10].

In this study, we identified 13 candidate regulatory targets of the TGF-β signaling pathway through a kinome-wide CRISPR knockout screen in HeLa cells. Among these, the well-known hallmark genes *TGFBR1* and *TGFBR2* ranked as the top two candidate targets. *OXSR1* and *EXOSC10* were ranked third and fourth as positive candidate targets, respectively, with *EXOSC10* being a novel finding. Importantly, we found that reduced expression of *OXSR1* and *EXOSC10* using CRISPR knockout and RNAi technology effectively down-regulated the TGF-β signaling pathway in HeLa and HaCaT cells, providing new insights of TGF-β signaling.

## 2 Material and methods

### 2.1 Cell culture

HeLa and HaCaT cells was obtained from the National Infrastructure of Cell Line Resource (Beijing, China) and is authenticated through short tandem repeat (STR) profiling. HeLa, HaCaT and HEK293T cells were cultured in Dulbecco’ modification Eagle’ Medium (DMEM, Corning) supplemented with 10 % FBS (Gibco) and 1 % penicillin-streptomycin (Gibco), and maintained at 37 °C with 5 % CO_2_.

### 2.2 Substances

Recombinant human TGF-β1 (240-B-002) was purchased from RD systems.

### 2.3 Lentivirus production

HEK293T cells with 80–90% confluent were co-transfected with Human kinome CRISPR knockout library (Brunello, Addgene Cat# 1000000083), psPax2 (Addgene, Cat# 12260, RRID: Addgene_12260), and pMD2.G (Addgene, Cat# 12259, RRID: Addgene_12259) at a ratio of 7: 5: 2 using Neofect DNA transfection reagent (Neofect, TF201201) following the manufacturer’s instructions. The culture media was replaced with viral production medium (Lonza) at 16–20 h post-transfection. The viral supernatant was then collected at 24 and 48 h after replacement, filtered with 0.45 μm filters (Millipore, Millipore SteriCup 250 mL), and stored at −80 °C.

### 2.4 Kinome-wide CRISPR-Cas9 knockout screen, fluorescent cell staining and FACS

The lentivirus of kinome library was transduced into HeLa cells at multiplicity of infection (MOI) about 0.3, and about 1000 coverage of the library sgRNAs. After 48 h of infection, the minimal lethal dose of puromycin (Thermo Fisher Scientific) was added to select for the transduced cells. Following 48 h of puromycin selection, 2 × 10^7^ transduced cells were harvested as pre-screening cells (T0), while the remaining cells were cultured for 7 days. At this point, some cells were collected as pre-sorting cells (T7), while others were stimulated with TGF-β1 for 1 h and then harvested for fluorescent cell staining.

The fluorescent cell staining was performed as described previously [11]. Briefly, cells was washed with PBS, fixed in 4% formaldehyde for 10 min at room temperature, washed with PBS, and then stained with Phospho-Smad2 Antibody (Cell Signaling Technology, Cat# 3104, RRID: AB_390732) in PBS containing 0.1% saponin for 30 min on ice. Finally, the cells was washed with PBS for FACS.

FACS sorting was performed using the SH800 Fully Automated Cell Sorter (Sony). HeLa cells were sorted into two bins based on P-Smad2 expression levels. The bins containing cells with low or high P-Smad2 expression was collected to identify positive and negative regulators of TGF-β signaling, designated as TGF^lo^ and TGF^hi^, respectively.

### 2.5 DNA purification, PCR library construction, and high throughput sequencing

The genomic DNA (gRNA) of T0, T7, TGF^hi^ and TGF^lo^ cells was extracted using Blood & Cell Culture DNA Maxi Kit (QIAGEN) following the manufacturer’s instruction. Library preparation for sequencing was performed as previously described [12]. In brief, two rounds of PCR was performed using the Next High Fidelity 2 × PCR Master Mix (New England Biolabs) to amplify sgRNA flanking regions and attach Illumina index and adaptor. The final PCR products were purified from the gel using Zymo Midi Purification Kit (Zymo) and sequenced with The Hiseq X10 platform (Illumina). The primer sequences used are as follows:

First-round forward: 5′-AATGGACTATCATATGCTTACCGTAACTTGAAAGTATTTCG-3’;

First-round reverse: 5′-GTGACTGGAGTTCAGACGTGTGCTCTTCCGATCTACTGACGGGCACCGGAGCCAATTCC-3’;

Second-round forward: 5′-AATGATACGGCGACCACCGAGATCTACACTCTTTCCCTACACGACGCTCTTCCGATCTTCTTGTGGAAAGGACGAAACACCG-3’;

Second-round reverse: 5′-CAAGCAGAAGACGGCATACGAGATNNNNNNGTGACTGGAGTTCAGACGTG-3’ (NNNNNN stands for 6bp index).

### 2.6 Analysis of CRISPR screen data

The CRISPR screen data was analyzed as described previously [12]. In brief, the sequencing data was processed using Fastx_barcode_splitter.pl command of FASTX-Toolkit software (version 0.0.14). Subsequently, FASTX-Toolkit software was used to extract 20 bp sgRNA sequences. Bowtie software (version 1.2.2) [13] was utilized to align sgRNA sequences to the kinome-wide library, allowing single nucleotide mismatch. Gene beta score was calculated using Maximum-likelihood estimation (MLE) command of Model-based analysis of genome-wide CRISPR/Cas9 knockout (MAGeCK) software (version 0.5.9.2) [14]. The Metascape database (version 3.5) [15] was utilized to identify pathway enrichment associated with the essential genes, which provides a comprehensive analysis of gene sets. Protein-protein interaction (PPI) analysis was performed using STRING database (version 12.0) [16].

### 2.7 siRNA transfection and TGF-β1 treatment

HeLa cells were transfected with *OXSR1* or *EXOSC10* siRNA (at a final concentration of 20 nM, RiboBio) using Lipofectamine RNAiMax reagent (Invitrogen). After 48 h, the cells were treated with 2 ng/ml TGF-β1 for 0 and 1 h, followed by qRT-PCR and western blot analysis. The siRNA sequences used in this study were as follows:

siOXSR1-#1: 5′-GCAGCAAUUUCACAACUCA-3’;

siOXSR1-#2: 5′-GCACCAACCAUUUCUGAAA-3’;

siDXSR1-#3: 5′-GGAUCAGGUUCACAAGAAA-3′

siEXOSC10-#1: 5′-GCAGAGUAAUGCAGUACCA-3′

siEXOSC10-#2: 5′-GUUUGCACAUCCUUAUCAA-3′

siEXOSC10-#3: 5′-GAAGGCAGCUGAGCAAACA-3′

### 2.8 CRISPR knockout and TGF-β1 treatment

The lentiCRISPR v2 plasmid (Addgene, Cat# 52961, RRID: Addgene_52961) ligated with non-targeting (NT), *OXSR1*, *EXOSC10*, *TGFBR1*, and *TGFBR2* sgRNAs were transfected into HEK293T cells using Neofect DNA transfection reagent to produce lentivirus. The lentivirus were transduced into HeLa and HaCaT cells. After 48 h, the transduced cells was selected with puromycin and treated with TGF-β1 for 0, 1, 4 and 12 h, followed by qRT-PCR and western blot analysis. The sgRNA sequences used in this study were as follows:

NT sgRNA: 5′-CTGAAGGTGTCTGGCAGAGC-3’;

*OXSR1* sgRNA: 5′-CCAACAAGGGGTGCCAACAA-3’;

EXOSC10 sgRNA: 5′-AGGCTGGCTGACCTTAACGA-3’;

*TGFBR1* sgRNA: 5′-ATTGTGTTACAAGAAAGCAT-3’;

*TGFBR2* sgRNA: 5′-ACCTACAGGAGTACCTGACG-3’.

### 2.9 qRT-PCR

Total RNA was extracted using TRIzol (Ambion) and the RevertAid First Strand cDNA Synthesis Kit (Thermo Fisher Scientific) was used to reverse transcribe 1 μg of total RNA into cDNA using according to the manufacturer’s instructions. LightCycler 480 SYBR Green I master (Roche) was used to perform quantitative reverse transcription PCR (qRT-PCR). The following primers were used:

*GAPDH* forward primer: 5′-TGCACCACCAACTGCTTAGC-3’;

*GAPDH* reverse primer: 5′-GGCATGGACTGTGGTCATGAG-3’;

*OXSR1* forward primer: 5′- ATCTACCATTGCTACGATACT-3’;

*OXSR1* reverse primer: 5′-ATCACCACCAGTTGCTAA-3’;

*EXOSC10* forward primer: 5′-GGGATGACACCCATTACCTGCTA-3’;

*EXOSC10* reverse primer: 5′-CATCCGTGAAGATAGGTTTGATG-3’;

*CTGF* forward primer: 5′-CTGCAGGCTAGAGAAGCAGAG-3’;

*CTGF* reverse primer: 5′-GATGCACTTTTTGCCCTTCT-3’;

*PAI-1* forward primer: 5′-ATTCAAGCAGCTATGGGATTCAA-3’;

*PAI-1* reverse primer: 5′-CTGGACGAAGATCGCGTCTG-3’;

*FN* forward primer: 5′-ACTGTACATGCTTCGGTCAG-3’;

*FN* reverse primer: 5′-AGTCTCTGAATCCTGGCATTG-3’;

*p21* forward primer: 5′-AGACCAGCATGACAGATT-3’;

*p21* reverse primer: 5′-AGGCAGAAGATGTAGAGC-3’.

### 2.10 Western blot

The Western Blot was performed as described previously [17]. Briefly, cells were lysed using RIPA lysis buffer (Beyotime Biotechnology), supplemented with protease (cOmplete, Roche) and phosphatase (PhosSTOP, Roche) inhibitor cocktail. Protein quantification was conducted using BCA method (Applygen Technologies). The prepared protein samples were loaded, electrophoresed, transferred, blocked, incubated with primary antibodies and secondary antibodies, and detected using ECL. The antibodies used were the following: anti-phospho-SMAD2 (Cell Signaling Technology, Cat# 3104, RRID: AB_390732), anti-SMAD2/3 (Cell Signaling Technology, Cat# 5678, RRID: AB_10693547), anti-OXSR1 (Abclonal, Cat# A15126, RRID: AB_2762011), anti-EXOSC10 (Abclonal, Cat# A17519, RRID: AB_2769379), anti-TGFBR1 (Abcam, Cat# ab235578, RRID: AB_3095072), anti-TGFBR2 (Abcam, Cat# ab259360), anti-p21 Waf1/Cip1 (Cell Signaling Technology, Cat# 2947, RRID: AB_823586), anti-beta-actin (Cell Signaling Technology, Cat# 3700, RRID: AB_2242334), anti-phospho-SMAD3 (Cell Signaling Technology, Cat# 9520, RRID: AB_2193207), anti-GAPDH (ZSGB-Bio, Cat# TA-08, RRID: AB_2747414).

### 2.11 Statistical analysis

Statistical analysis was performed using GraphPad Prism 5. Two-tailed unpaired Student *t*-test was used to calculate the statistical significance between groups. ∗, *p* < 0.05; ∗∗, *p* < 0.01; ∗∗∗, *p* < 0.001.

## 3 Results

### 3.1 A kinome-wide CRISPR-Cas9 knockout screen in HeLa cells to identify the regulator of TGF-β signaling

**Fig. 1:**
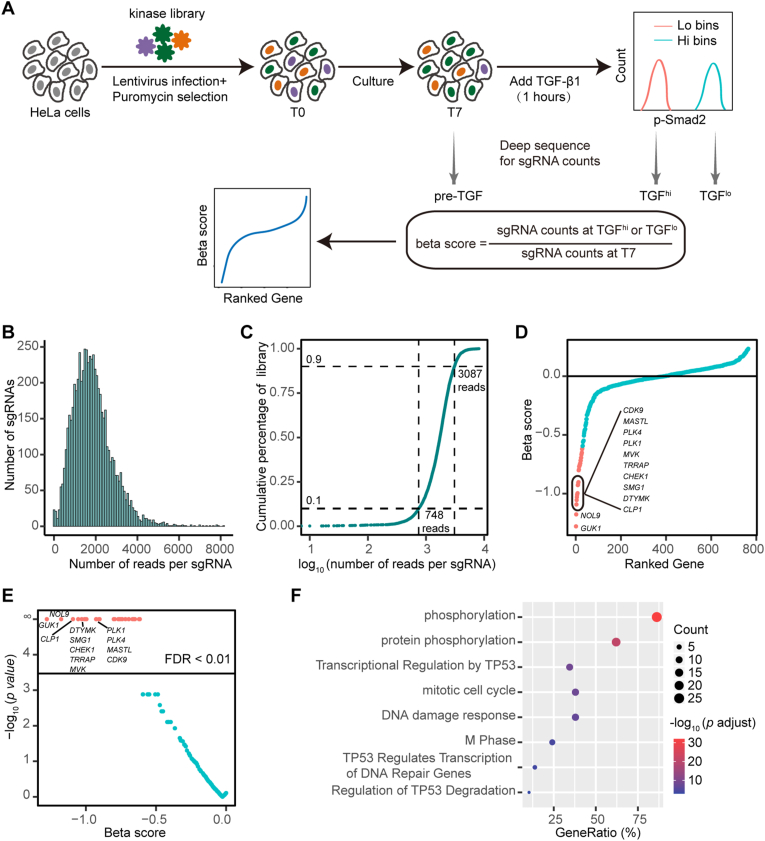
**Workflow and quality control of the kinome-wide CRISPR-Cas9 screen. A,** Workflow of CRISPR-Cas9 knockout screen using human kinome CRISPR knockout library (Brunello) in HeLa cells. **B** and **C**, Frequency histogram (**B**) and cumulative distribution (**C**) of the number of sequencing reads per sgRNA in T0 sample of the kinome-wide screen. **D** and **E**, Beta score was calculate using MAGeCK MLE algorithm for essential genes of HeLa cells (**D** and **E**). **F**, Pathway enrichment associated with the essential genes using Metascape database.

To systematically identify regulators of TGF-β signaling in protein kinases, we performed CRISPR-Cas9 screen in HeLa cells using human kinome-wide CRISPR-Cas9 knockout library (Brunello) [18], which consists of 6,104 unique sgRNAs target 763 human kinases, with 100 non-targeting sgRNAs as control. The CRISPR screen was performed with a multiplicity of infection (MOI) of approximately 0.3, for which the low MOI was chosen to ensure that each cell receives one sgRNA, maximizing the specificity of the knockout effect mediated by sgRNA (Fig. 1A). After 48 h of transfection, puromycin was added to select transfected cells for 48 h. At this point, 2 × 10^7^ cells were collected as pre-screening cells (T0) to assess the library integrity and uniformity in the samples after lentivirus transduction. The remaining cells were cultured for 7 days and collected as pre-sorting cells (T7). HeLa cells were stimulated with TGF-β1 for 1 h and TGF-β signaling active and inactive cells were sorted based on P-Smad2 expression levels (Fig. 1A). These sorted cells were collected as TGF^hi^ or TGF^lo^, respectively. We hypothesized that sgRNAs targeting positive regulators of TGF-β signaling would be enriched in TGF^lo^ compared to T7, while sgRNAs targeting negative regulators would be enriched in TGF^hi^ compared to T7. Additionally, sgRNAs targeting essential genes for HeLa cells would be reduced in T7 compared to T0.

### 3.2 Quality control of the kinome-wide CRISPR-knockout screen

To evaluate the initial quality of the screen, we first examined the sgRNA abundance in the T0 sample. Statistical analysis revealed that the number of sgRNA reads in the kinome-wide CRISPR-Cas9 library basically followed a normal distribution (Fig. 1B). Out of 6,204 sgRNAs in Brunello kinome library, only 8 of them had zero sequencing reads and all 763 human kinases could be targeted (Fig. 1B and C). The range of sgRNA reads for 10%–90% of T0 sample was between 748 and 3087 reads (Fig. 1C). These results demonstrated that the good integrity and uniformity of the Brunello kinome library after lentivirus transduction, confirming the reliability of the subsequent screen results.

To further assess the initial quality of the screen, we calculated the essential score (β score) of HeLa cells using maximum-likelihood estimation (MLE) module of model-based analysis of genome-wide CRISPR/Cas9 knockout (MAGeCK) [14], identifying 29 candidate essential genes with β scores > 0 and false discovery rate (FDR) < 1% (Fig. 1D and E). As anticipated, these candidate essential genes were mainly enriched in pathways associated with core essential genes, such as phosphorylation, TP53-regulated transcription, cell cycle and DNA damage response (Fig. 1F), which further supports the reliability of the screen.

### 3.3 Candidate hits for regulator of TGF-β signaling identified by kinome-wide CRISPR-Cas9 knockout screen

**Fig. 2:**
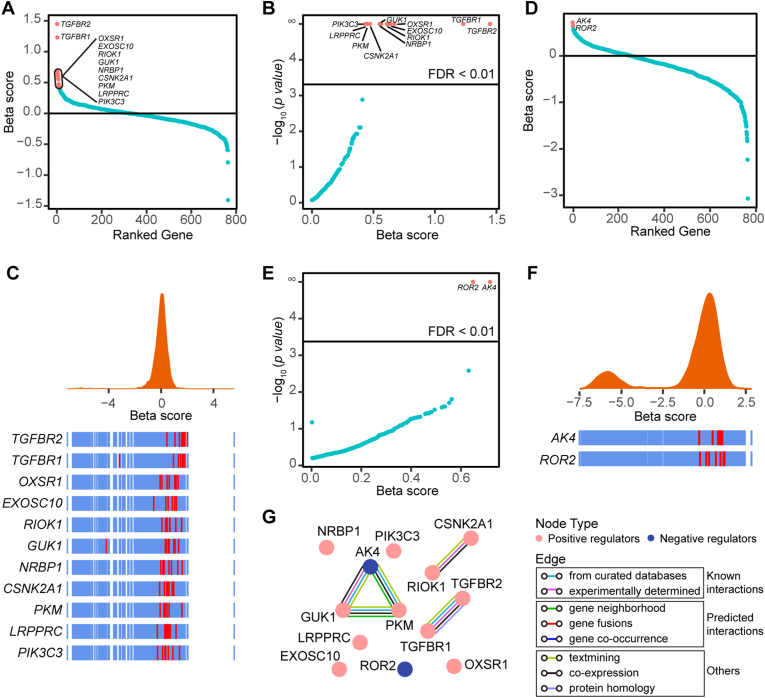
Kinome-wide screen outcomes. A and B, Beta score of positive regulators was calculated using MAGeCK MLE algorithm (A and B). Candidate genes are marked red. C, Frequency histograms of sgRNA beta scores showing enrichment of positive candidate genes in HeLa cells. Lines representing the beta scores of individual sgRNAs targeting candidate genes are marked red. D and E, Beta score of negative regulators was calculated using MAGeCK MLE algorithm (D and E). Candidate genes are marked red. F, Frequency histograms of sgRNA beta scores showing enrichment of negative candidate genes in HeLa cells. Lines representing the beta scores of individual sgRNAs targeting candidate genes are marked red. G, Protein-protein interaction analysis of the candidate genes. (For interpretation of the references to colour in this figure legend, the reader is referred to the Web version of this article.)

Next, we employed MAGeCK MLE analysis to identify positive and negative regulators of the TGF-β signaling pathway in the low-expression and high-expression groups. The candidate targets for positive and negative regulation were identified with the criteria of β score > 0 and false discovery rate (FDR) < 1 %. A total of 11 candidate targets for positive regulator were identified. Notably, these targets included hallmark members of TGF-β signaling pathway, *TGFBR2* (rank 1) and *TGFBR1* (rank 2), as well as previously reported gene, *OSXR1* [19] (rank 3). Additionally, we discovered several novel candidate genes, including *EXOSC10*, *RIOK1*, *GUK1*, *NRBP1*, *CSNK2A1*, *PKM*, *LRPPRC*, and *PIK3C3* (Fig. 2A–C, Table 1). Furthermore, we identified 2 candidate targets for negative regulation including *AK4* and *ROR2* (Fig. 2D–F, Table 1). Although our screening is theoretically capable of detecting both positive and negative regulators of the TGF-β signaling pathway, it may exhibit reduced sensitivity in identifying negative regulators due to the high baseline TGF-β signaling activity and a limited dynamic range that can further enhance the TGF-β signaling pathway.

**Table 1:** The candidate genes of TGF-β signaling in kinome-wide CRISPR screen.

Gene symbol	Beta score	*p value*	fdr	Class
*TGFBR2*	1.4483	0	0	Positive candidate
*TGFBR1*	1.2302	0	0	Positive candidate
*OXSR1*	0.67059	0	0	Positive candidate
*EXOSC10*	0.63531	0	0	Positive candidate
*RIOK1*	0.61042	0	0	Positive candidate
*GUK1*	0.5621	0	0	Positive candidate
*NRBP1*	0.55055	0	0	Positive candidate
*CSNK2A1*	0.47144	0	0	Positive candidate
*PKM*	0.44655	0	0	Positive candidate
*LRPPRC*	0.44407	0	0	Positive candidate
*PIK3C3*	0.43353	0	0	Positive candidate
*AK4*	0.7172	0	0	Negative candidate
*ROR2*	0.64843	0	0	Negative candidate

To further explore the mechanism of the hits for regulation of TGF-β signaling, protein-protein interaction (PPI) analysis was performed with positive and negative candidate hits. It revealed this network has 5 edges, containing *TGFBR2* with *TGFBR1, RIOK1* with *CSNK2A1*, *GUK1* with *PKM*, *GUK1* with *AK4*, *and PKM* with *AK4* (Fig. 2G).

### 3.4 Candidate regulators of TGF-β signaling validated in HeLa cells

**Fig. 3:**
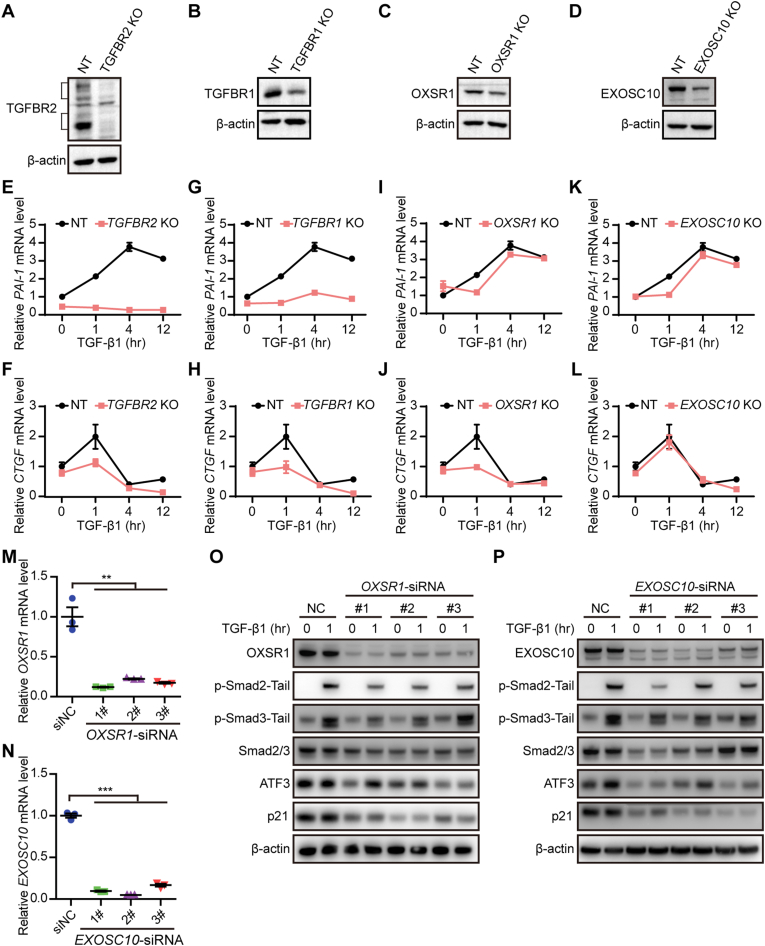
Functional validation of *candidate* hits in HeLa cells. A-D, Western Blot analysis of HeLa cells transduced with *TGFBR2* (A)*, TGFBR1* (B), *OXSR1* (C), or *EXOSC10* (D) sgRNAs. E-L, qRT-PCR analysis of HeLa cells after transfection with *TGFBR2* (E and F) or *TGFBR1* (G and H)*, OXSR1* (I and J), or *EXOSC10* (K and L) sgRNAs. M and N, qRT-PCR analysis was used to detect the *OXSR1* (M) or *EXOSC10* (N) gene expression of HeLa cells after transfection with *EXOSC10* or *OXSR1* siRNAs. HeLa cells transfected with siRNA for 24 h were collected for qRT-PCR. O and P, Western Blot analysis of EXOSC10 or OSXR1, p-Smad2-Tail, p-Smad2-Tail, Smad2/3, ATF3, and p21 in HeLa cells after transfection with *OXSR1* (O) or *EXOSC10* (P) siRNAs treated with TGF-β1. Unpaired and two-tailed t-tests were used to determine *P* values. ∗, *p* < 0.05; ∗∗, *p* < 0.01; ∗∗∗, *p* < 0.001.

To validate the role of top 4 candidate regulators in the TGF-β signaling pathway, CRISPR knockout technology was utilized to perform gene knockout in HeLa cells. Western Blot analysis demonstrated the effectiveness of *TGFBR2, TGFBR1, OXSR1* or *EXOSC10* sgRNAs in reducing protein levels of *TGFBR2, TGFBR1, OXSR1* or *EXOSC10* (Fig. 3A–D). qRT-PCR analysis revealed that the down-regulation of *TGFBR2, TGFBR1, OXSR1* or *EXOSC10* resulted in decreased expressions of plasminogen activator inhibitor type 1 (*PAI-1*) and connective tissue growth factor (*CTGF*) (Fig. 3E–L), which are known target genes of TGF-β signaling pathway [20,21]. Moreover, the down-regulation of *OXSR1* or *EXOSC10* using RNAi technology also resulted in decreased expressions of the phospho-Smad2 and phospho-Smad3, as well as, Smad2/3, cyclin-dependent kinase inhibitor 1A (p21), and ATF3, which are also known TGF-β target genes [[22], [23], [24]] (Fig. 3M–P).

### 3.5 *OXSR1 and EXOSC10* knockout suppressed the TGF-β signaling in HaCaT cells

**Fig. 4:**
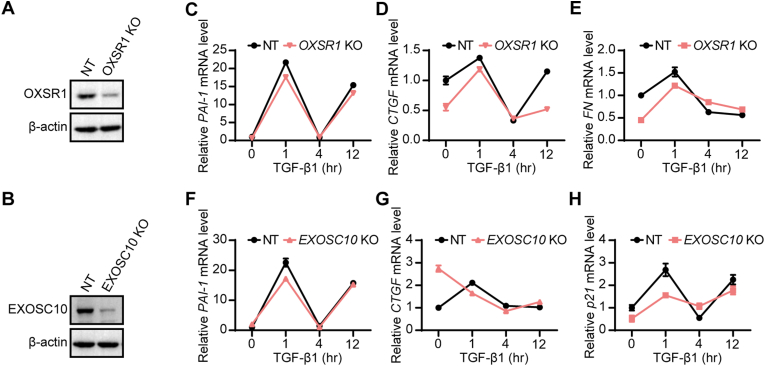
**Functional validation of *OXSR1 and EXOSC10* genes in HaCaT cells. A** and **B**, Western Blot analysis of HaCaT cells transduced with *OXSR1* (**A**) or *EXOSC10* (**B**) sgRNAs. **C**–**H**, qRT-PCR analysis of HaCaT cells after transfection with *OXSR1* (**C**–**E**) or *EXOSC10* (**F**–**H**) sgRNAs.

In addition to the hallmark members of TGF-β signaling pathway*, OSXR1* and *EXOSC10* have emerged as the top candidate genes. To further validate the role of *OXSR1* and *EXOSC10* in the TGF-β signaling pathway, CRISPR knockout technology was utilized in HaCaT cells. After the down-regulation of *OXSR1* or *EXOSC10* in HaCaT cells, we observed a similar decrease in the expression of TGF-β target genes [20,21,23,25], including P*AI-1*, *CTGF*, fibronectin (*FN*) or *p21*(Fig. 4A–H). These findings indicate that the reduced expression of *OXSR1* or *EXOSC10* can effectively down-regulate *the* TGF-β signaling pathway.

## 4 Discussion

In this work, we performed CRISPR-Cas9 knockout screen to systematically identify regulators of TGF-β signaling in protein kinases, with a total of 13 candidate targets were identified, containing 11 positive regulators and 2 negative regulators.

Interestingly, *TGFBR2* and *TGFBR1*, identified as the top two hits of positive regulators in our screen, have been reported in multiple studies to interact with TGF-β, forming ligand-receptor complexes that phosphorylate downstream proteins such as SMAD, thereby mediating TGF-β signaling [26,27]. This further validates the reliability of our screen results and underscores the pivotal role of *TGFBR2* and *TGFBR1* in the TGF-β signaling pathway.

Furthermore, the third and fourth-ranked positive regulators, *OXSR1* and *EXOSC10*, were identified using CRISPR knockout and RNAi technology in HeLa and HaCaT cells. Specifically, *OXSR1* has been previously reported to mediate the TGF-β signaling pathway in breast cancer, promoting epithelial-mesenchymal transition (EMT) and metastasis through the phosphorylation of Smad2/3 [19], *EXOSC10* is a novel candidate factor that had not been identified before. As an exosome catalytic subunit, *EXOSC10* is involved in RNA processing and degradation, as well as in the negative regulation of telomere by degrading telomerase RNA components and participating in DNA double-strand break repair [28,29]. Our findings suggest that the reduced expression of *OXSR1* and *EXOSC10* can effectively down-regulate the TGF-β signaling pathway, indicating their positive regulatory roles in the TGF-β signaling pathway.

Protein-protein interaction (PPI) network analysis revealed 13 candidate hits clustered into 3 groups. The one group is *TGFBR2* and *TGFBR1.* The second group is *RIOK1* and *CSNK2A1*. Both *RIOK1* [30,31] and *CSNK2A1* [32,33] are involved in the regulation of PI3K-AKT pathway, promoting cancer cell migration and invision through the PI3K-AKT signaling pathway, indicating an interaction between the PI3K-AKT and TGF-β pathways. The third group is *GUK1*, *PKM* and *AK4*. Previous studies showed that *AK4* and *PKM* are related to HIF-1α pathway, which regulates TGF-β pathway [[34], [35], [36]]. While *GUK1* has not been previously reported in this context, the PPI analysis suggests it may also be involved in the HIF-1α pathway and these 3 genes (*GUK1*, *PKM*, and *AK4*) appear to function through the HIF-1α pathway.

In summary, based on kinome-wide CRISPR knockout screen, we identified 13 candidate regulators of the TGF-β signaling pathway, including known regulators such as *TGFBR2* and *TGFBR1*, as well as novel regulators such as *EXOSC10*, providing new insights on the regulation of the TGF-β signaling.

## CRediT authorship contribution statement

**Dingding Wang:** Writing – original draft, Visualization, Funding acquisition, Data curation. **Xinhao Zhang:** Data curation. **Jianxun Guo:** Data curation. **Weijia Liu:** Data curation. **Yanchi Zhou:** Writing – original draft, Data curation. **Renxian Wang:** Writing – original draft, Funding acquisition.

## Ethics statement

This study did not involve human or animal subjects, and thus, no ethical approval was required.

## Funding

This work was supported by the 10.13039/501100012166National Key Research and Development Program of China (2023YFC2507600), 10.13039/501100001809National Natural Science Foundation of China (52173275), Cultivation Project of Natural Science Foundation, Beijing Jishuitan Hospital, 10.13039/501100002799Capital Medical University (ZR-202410), Beijing Municipal Public Welfare Development and Reform Pilot Project for Medical Research Institutes (JYY2023-11, JYY2023-8), 10.13039/501100005088Beijing Municipal Health Commission (BJRITO-RDP-2024).

## Declaration of competing interest

The authors declare no competing interests.

## Data Availability

The datasets generated and analyzed during the current study are available from the corresponding author on reasonable request.
